# Correlation Between Changes in Brand-Name Drug Prices and Patient Out-of-Pocket Costs

**DOI:** 10.1001/jamanetworkopen.2021.8816

**Published:** 2021-05-04

**Authors:** Benjamin N. Rome, William B. Feldman, Rishi J. Desai, Aaron S. Kesselheim

**Affiliations:** 1 Program On Regulation, Therapeutics, And Law, Division of Pharmacoepidemiology and Pharmacoeconomics, Department of Medicine, Brigham and Women’s Hospital, Boston, Massachusetts; 2 Harvard Medical School, Boston, Massachusetts; 3 Division of Pulmonary and Critical Care Medicine, Department of Medicine, Brigham and Women’s Hospital, Boston, Massachusetts

## Abstract

**Importance:**

List prices set by manufacturers for brand-name prescription drugs in the US have been increasing faster than inflation, although confidential manufacturer rebates offset some of these increases. Most commercially insured patients pay at least some out-of-pocket costs for prescription drugs, and higher patient spending is associated with lower adherence and worse health outcomes.

**Objective:**

To examine whether price changes for brand-name drugs are correlated with changes in patient out-of-pocket spending and whether this association varies by insurance benefit design.

**Design, Setting, and Participants:**

A cohort study of 79 brand-name drugs with available pricing data from January 2015 to December 2017 was conducted, with data obtained from a national commercial insurance claims database.

**Exposures:**

Change in the list prices set by manufacturers and estimated net prices after rebates among non-Medicaid payers.

**Main Outcomes and Measures:**

Change in median out-of-pocket spending among all patients and stratified by insurance pharmacy benefit design, including high-deductible insurance plans and plans with any amount of deductibles or coinsurance.

**Results:**

Among 79 drugs, median increases were 16.7% (interquartile range [IQR], 13.6%-21.1%) for list prices, 5.4% (IQR, −3.9% to 11.7%) for net prices, and 3.5% (IQR, 1.4%-9.1%) for out-of-pocket spending from 2015 to 2017. Changes in list prices were correlated with changes in net prices (*r* = 0.34; *P* = .002). Overall, changes in out-of-pocket spending were not correlated with changes in list prices (*r* = 0.14; *P* = .22) or net prices (*r* = 0.04; *P* = .71). Among 53.7% of patients who paid any drug deductible or coinsurance, median out-of-pocket spending increased by 15.0%, and changes were moderately correlated with changes in list prices (*r* = 0.38; *P* = .001) but not net prices (*r* = 0.06; *P* = .62).

**Conclusions and Relevance:**

Some commercially insured patients who pay only prescription drug copayments appear to be insulated from increases in drug prices. However, more than half of patients pay deductibles or coinsurance and may experience substantial increases in out-of-pocket spending when drug prices increase. Among these patients, there was no evidence that manufacturer rebates to insurers are associated with patients’ out-of-pocket spending. Policies to rein in unregulated annual increases in list prices for brand-name drugs may have important consequences for patient out-of-pocket spending.

## Key Points

**Question:**

Among commercially insured patients with varying pharmacy benefit designs, are changes in prices for brand-name prescription drugs correlated with changes in patient out-of-pocket costs?

**Findings:**

In this cohort study of 79 brand-name drugs, there was no correlation between changes in drug prices and out-of-pocket spending. However, among the 57.3% of patients whose insurance required coinsurance or deductibles, changes in out-of-pocket spending were correlated with changes in list prices but not postrebate net prices.

**Meaning:**

Some commercially insured patients are insulated from paying more when drug list prices increase, but patients with deductibles and coinsurance pay more when manufacturers raise a drug’s list price and do not directly benefit from confidential rebates paid by manufacturers to insurers.

## Teaser

This cohort study examines the association between price changes for brand-name drugs and out-of-pocket spending by patients with different insurance plans.

## Introduction

Increasing prescription drug prices are a source of concern in the US. In 2018, the US spent $476 billion on prescription drugs, with 80% of spending on brand-name products.^1,2^ Brand-name manufacturers freely set list prices (akin to the sticker price for a car) for their drugs at whatever level they choose, and these prices increased by 9.1% per year on average over the past decade.^3^ For many drugs, manufacturers have offset increasing list prices by providing confidential rebates negotiated with pharmacy benefit managers and insurers.^4,5^ Although rebate levels vary greatly by drug class, some have estimated that despite continued year-over-year increases in list prices, growth in net prices after accounting for rebates has slowed or halted since 2015, particularly for certain drug classes.^1,2,3^

However, rebates negotiated by insurers are not necessarily reflected in the out-of-pocket prescription drug costs borne by patients. Patient spending at the pharmacy counter—including copayments, coinsurance, and deductibles—accounted for 14% of all US prescription drug spending in 2018.^2^ For patients in Medicare Part D plans, cost-sharing is typically higher and is often determined as a fixed percentage of a drug’s list price (25% of drug costs until patients reach the catastrophic coverage phase).^6^ As a result, Part D beneficiaries may be adversely affected by increasing list prices and may not benefit directly from rebates.^7^

Half of individuals in the US are insured by employer-based commercial insurance plans,^8^ which traditionally charge patients flat copayments for prescription drugs but have increasingly shifted costs to patients by adding deductibles and/or coinsurance.^9^ Yet few studies have examined the association between increasing list prices and patient out-of-pocket costs for commercially insured patients. One recent study noted that list prices for 14 top-selling drugs doubled from 2010 to 2016, while median out-of-pocket costs increased by 53%.^10^ Another study reported that, although insulin list prices more than doubled from 2006 to 2017, patient out-of-pocket spending was relatively stable over the same period.^11^ The latter study stratified patients based on whether they were in a high-deductible insurance plan, but to our knowledge, no study has systematically examined the relationship between drug prices and patient out-of-pocket costs stratified by commercial pharmacy benefit design.

Given the limited understanding of how prices affect patient out-of-pocket costs for commercially insured individuals, we examined the correlation between changes in list prices, net prices, and out-of-pocket spending among a cohort of brand-name drugs used by commercially insured patients from January 2015 to December 2017, overall and among patients with different insurance benefit designs.

## Methods

### Data Sources

In this cohort study, we identified the drug cohort and obtained list and net price estimates from SSR Health, which estimates prices and rebates for more than 1000 brand-name drugs manufactured by publicly traded companies.^12^ We measured median patient out-of-pocket spending using claims data from a national sample of approximately 30 million patients with employer-sponsored commercial insurance at any given time (MarketScan; IBM Truven Health Analytics).

We obtained approval from the Mass General Brigham Institutional Review Board to use deidentified claims data for this study. This study followed the Strengthening the Reporting of Observational Studies in Epidemiology (STROBE) reporting guideline for cohort studies.

### Drug Cohort

SSR Health provides data for drugs with at least 1 net price estimate from 2007 to 2019, but most drugs do not have estimates for the entire period. We included only drugs with continuous net price estimates in each quarter from 2015 to 2017 and drugs with at least 1000 units reported per quarter to minimize the instability of net price estimates. We excluded drugs administered by clinicians (eg, vaccines, intravenous preparations) because pharmacy claims may not accurately capture out-of-pocket spending for these products. We also excluded brand-name drugs with generic competition before January 2018 because insurers may increase patient cost-sharing for brand-name products after generic versions are released. Generic entry date was obtained from SSR Health, supplemented by a review of when the first generic National Drug Codes were listed in a compendium of all drug products published by First Databank.^13^ We also excluded contraceptives, which all commercial insurers have generally been required to cover without cost-sharing since passage of the Affordable Care Act.^14^

Pricing estimates in SSR Health are given for an entire drug product, representing a weighted average of unit prices across all formulations and dosage forms. A given product may have variable definitions of units (eg, tablets vs milliliters of solution) and different prices per unit. Consequently, variation in the relative use of different dosage forms may result in changes in the product’s unit price over time that do not reflect variations in the price of the drug. To narrowly focus on the association between a drug’s price and out-of-pocket costs, we included only drug products that had the same unit price across all dosage forms. In addition, we included only drugs for which we could identify a standard number of units per 30-day supply based on the dosing guidelines on the drug’s US Food and Drug Administration–approved labeling. For drugs with prices that vary based on the dose prescribed, out-of-pocket spending may be influenced both by price and dose, and our aim was to focus solely on the association between price and out-of-pocket spending.

For each drug included in the cohort, we used the US Food and Drug Administration–approved labeling to determine the drug’s route of administration and therapeutic category. We also estimated annual US revenue in 2015 and 2017 as a product of net price multiplied by estimated use among all patients in the US from SSR Health.

### Drug List and Net Prices

We obtained quarterly wholesale acquisition cost (ie, the list price set by manufacturers) and net prices from SSR Health, which estimates net prices by comparing publicly reported manufacturer revenue with estimates of total number of units sold in the US.^12^ These estimates approximate the average US price per unit after manufacturer rebates and other price concessions. We relied on SSR Health’s non-Medicaid rebate estimates because Medicaid rebates are statutory and usually higher than those for Medicare or commercial plans.^15,16^

Because drug sales volume to wholesalers can vary over a calendar year (eg, the drug can be stocked in January to last several months), we calculated annual averages for list and non-Medicaid net prices in 2015 and 2017, weighting each quarter equally. All annual average prices were converted to 2017 US dollars using the Consumer Price Index for all urban consumers.^17^ We also converted unit prices to the price for a typical 30-day supply of the drug based on the dosing instructions in the US Food and Drug Administration–approved labeling information. For drugs typically dispensed for 1-time use (eg, bowel preparation regimens), we converted to the price for a single use.

In some cases, SSR Health’s quarterly net price estimates exceeded list prices, usually because the number of units sold may be underreported.^3^ When net price exceeded list price in a given quarter, we presumed rebates of 0, so that the net price was equal to the list price for that quarter. Owing to unequal distribution of drugs throughout the year, a negative rebate in one quarter might offset a high rebate in a surrounding quarter. Therefore, assuming 0 rebates in these cases may lead to underestimates of annual average net prices. We also performed a sensitivity analysis in which we excluded drugs with a negative rebate estimate in any quarter.

### Out-of-Pocket Spending

For each brand-name drug, we identified separate cohorts of patients in 2015 and 2017 with at least 1 claim for the drug. We excluded patients with claims paid under capitated payment models because cost data for these claims are not reliably recorded.

We calculated the annual sum of out-of-pocket costs (copayments, coinsurance, and deductibles) paid across all filled prescriptions for the drug of interest in each calendar year. We also summed the annual quantity of drug prescribed (the number of units), which we converted to 30-day supplies to calculate each patient’s average annual out-of-pocket spending per 30-day supply (or 1-time use). We then calculated the median out-of-pocket spending among all users of each drug in each year, adjusted to 2017 US dollars. To ensure that unit prices were measured similarly in SSR Health and claims data, we calculated median total drug cost per unit in the claims data (including patient and payer components) and excluded drugs if this cost differed from the drug’s list price by more than 10%.

### Statistical Analysis

For each drug, we calculated the relative change in inflation-adjusted list price, non-Medicaid net price, and median out-of-pocket spending from 2015 to 2017 (eg, 2017 price – 2015 price / 2015 price). We calculated Pearson correlation coefficients between relative changes in drug prices and out-of-pocket spending to identify significant associations (*r* ≠ 0) at a 2-sided significance level of *P* < .05.

To account for varying associations between changes in drug prices and out-of-pocket spending among patients with different insurance benefit designs, we performed stratified analyses in which we correlated drug prices with median out-of-pocket spending among patients in high-deductible insurance plans and those who paid a deductible and/or coinsurance toward any prescription drug claim in the given year.

In the primary analysis, we weighted each drug based on its use, defined as the sum of 30-day supplies dispensed in the US in 2015 and 2017, obtained from SSR Health. The weighting was performed to ensure that our results were reflective of the highest-used drugs with the greatest public policy importance. In a secondary analysis, we calculated unweighted estimates. To test for bias resulting from our assumption of 0 rebates in any quarter when net price estimates exceeded list prices, we performed a sensitivity analysis that excluded the 21 drugs for which estimated net prices exceeded list price in any quarter. Analyses were performed using SAS Software, version 9.4 (SAS Institute Inc).

## Results

Of 1117 brand-name products with any SSR Health net price estimates from 2007 to 2019, 541 drugs had continuous net price estimates in each quarter from 2015 to 2017. Ultimately, 79 self-administered brand-name drugs without generic competition met the entry criteria for the cohort (eFigure 1 in the Supplement). A list of included drugs is provided in eTable 1 in the Supplement.

Of the 79 drugs in our cohort, 64 (81%) were administered orally, 8 (10%) by injection, 3 (4%) by inhalation, and the remaining 4 (5%) via topical, rectal, or ophthalmic routes. The most common therapeutic categories were oncologic (11 [14%]), antimicrobial (9 [11%]), endocrine/diabetes (9 [11%]), neurologic (9 [11%]), cardiovascular (8 [10%]), and gastrointestinal (8 [10%]).

Median US annual revenue per drug was $330 million (interquartile range [IQR], $120 million-$706 million) in 2015 and $401 million (IQR, $120 million-$888 million) in 2017. Total revenue for all 79 drugs was $63 billion in 2015 and $67 billion in 2017. In 2017, 18 drugs (23%) had annual revenue exceeding $1 billion.

### List and Net Prices

Median list prices per 30-day supply increased from $333 (IQR, $229-$346) in 2015 to $386 (IQR, $271-$390) in 2017, with a median change in list price of 16.7% (IQR, 13.6%-21.1%) (Table 1). The unweighted median list prices were higher, but the percent change was similar at 17.2% (IQR, 12.3%-23.3%). List prices increased for all but 4 drugs; these drugs each had a 3.3% decrease in price from 2015 to 2017 related to inflation (ie, no change in nominal list price).

**Table 1.** Drug Prices and Out-of-Pocket Spending, 2015 to 2017^a^

Variable	List price per 30-d supply			Net price per 30-d supply			Out-of-pocket spending per 30-d supply		
	2015, $	2017, $	Change, %	2015, $	2017, $	Change, %	2015, $	2017, $	Change, %
All 79 drugs, median (IQR)									
Weighted by use	333 (229-346)	386 (271-390)	16.7 (13.6-21.1)	173 (139-223)	166 (144-234)	5.4 (−3.9 to 11.7)	29 (26-43)	30 (26-45)	3.5 (1.4-9.1)
Unweighted	691 (294-6024)	900 (350-7240)	17.2 (12.3-23.3)	492 (187-5315)	489 (200-5845)	7.5 (−1.9 to 18.6)	36 (31-47)	40 (30-50)	4.9 (−3.3 to 14.7)
10 Drugs with highest use^b^									
Sitagliptin (Januvia)	346	390	12.8	173	144	−17.2	26	26	1.4
Lisdexamfetamine (Vyvanse)	229	271	18.2	140	153	11.7	46	50	9.1
Rivaroxaban (Xarelto)	333	386	15.6	217	208	−3.9	29	30	1.8
Nebivolol (Bystolic)	98	120	22.0	65	69	6.3	22	25	11.5
Apixaban (Eliquis)	333	388	16.7	223	234	5.4	27	28	3.3
Dexlansoprazole (Dexilant)	221	255	15.6	88	88	0.1	41	45	8.3
Solifenacin (Vesicare)	254	317	24.8	115	107	−5.9	26	27	3.7
Adalimumab (Humira)	3494	4649	33.0	2687	3239	24.0	43	56	28.9
Linagliptin (Tradjenta)	343	381	11.2	125	126	1.6	22	25	11.5
Linaclotide (Linzess)	294	353	20.2	194	194	0.5	36	35	−3.3

Abbreviation: IQR, interquartile range.

^a^ All prices are in 2017 US dollars and represent the cost for a 30-day drug supply.

^b^ Drugs are listed in descending order of use.

**Figure 1.**
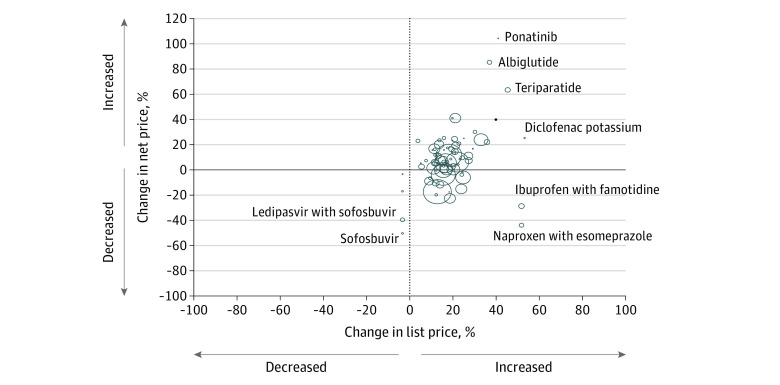
Correlation Between Changes in List and Net Prices Each bubble represents 1 drug, with the area of the bubble proportional to the number of 30-day supplies of the drug dispensed in the US in 2015 and 2017. The x-axis represents the relative change in list price and the y-axis the change in net-price from 2015 to 2017. Outliers are labeled by generic name; corresponding brand names can be found in eTable 1 in the Supplement.

The median net price per 30-day drug supply was $173 (IQR, $139-$223) in 2015 and $166 (IQR, $144-$234) in 2017. The median change in net price was 5.4% (IQR, −3.9% to 11.7%). Unweighted results revealed a similar median increase in net price of 7.5% (IQR, −1.9% to 18.6%). Net prices decreased for 21 drugs (27%) and increased for 58 drugs (73%) (Table 1). Changes in list and net prices were positively correlated in weighted (*r* = 0.34; *P* = .002) (Figure 1) and unweighted (*r* = 0.39; *P* < .001) analyses.

### Out-of-Pocket Spending

The median number of commercially insured patients in our data set who used each drug was 4925 (IQR, 1281-16 266) in 2015 and 4078 (IQR, 772-19 907) in 2017. The sum of patients included in the median out-of-pocket spending estimates was 1.4 million in each year, although some patients may have used more than 1 drug in the cohort in a given year.

Median out-of-pocket spending for a 30-day drug supply was $29 (IQR, $26-$43) in 2015 and $30 (IQR, $26-$45) in 2017, with a median change from 2015 to 2017 of 3.5% (IQR, 1.4%-9.1%). Results were similar in unweighted analyses.

**Figure 2.**
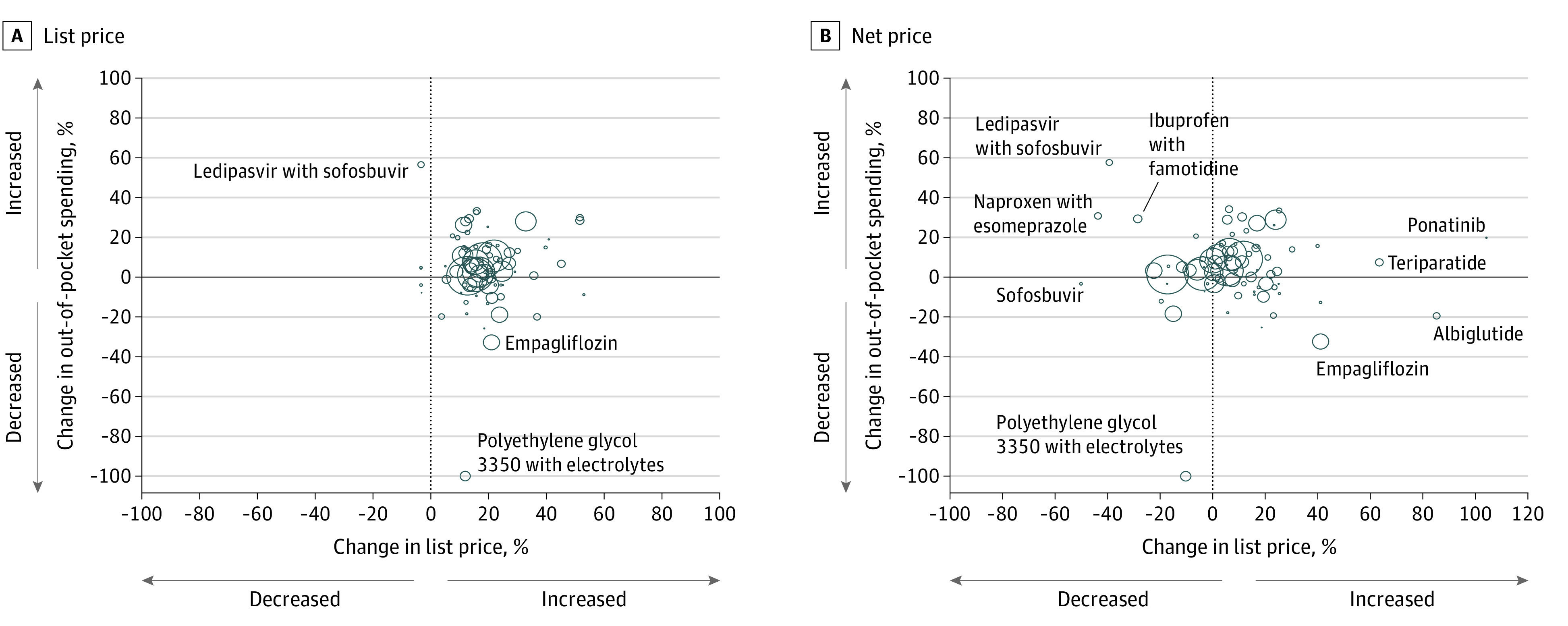
Correlation Between Changes in Drug Prices and Out-of-Pocket Spending Each bubble represents 1 drug, with the area of the bubble proportional to the number of 30-day supplies of the drug dispensed in the US in 2015 and 2017. The x-axis represents the change in list (A) or net (B) price, and the y-axis represents the change in out-of-pocket spending from 2015 to 2017. Outliers are labeled by generic name; corresponding brand names can be found in eTable 1 in the Supplement. One drug, polyethylene glycol 3350/electrolytes (MoviPrep), had a median out-of-pocket spending of $0 in 2017, resulting in a 100% decrease.

In 2017, 16.7% of patients in our cohort were enrolled in high-deductible health plans and 53.7% paid either a deductible or coinsurance (Table 2). Out-of-pocket spending was higher among patients in high-deductible health plans (median, $56 in 2017; IQR, $43-$65) than among those not in high-deductible health plans (median, $28; IQR, $25-$42) and among those who paid any deductible or coinsurance (median, $40 in 2017; IQR, $30-$54) compared with those with no deductible or coinsurance (median, $25; IQR, $23-$35). The change in out-of-pocket spending from 2015 to 2017 was higher among patients with any deductible or coinsurance (median, 15.0% vs −3.3%). In weighted analyses, there was no correlation between changes in out-of-pocket spending and changes in list price (*r* = 0.14; *P* = .22) or net price (*r* = 0.04; *P* = .71) (Figure 2).

**Table 2.** Changes in OOP Spending and Correlation With Changes in Drug Prices^a^

Subgroup^b^	2015		2017		% Change in OOP spending, 2015 to 2017, weighted median (IQR)	Weighted correlation with change in list price		Weighted correlation with change in net price	
	Patients, %	OOP spending, weighted median (IQR)	Patients, %	OOP spending, weighted median (IQR)		*r*	*P* value	*r*	*P* value
All patients	100	29 (26 to 43)	100	30 (26 to 45)	3.5 (1.4 to 9.1)	0.14	.22	0.04	.71
High-deductible plan									
Yes	12.7	55 (51 to 66)	16.7	56 (43 to 65)	−3.3 (−6.2 to 8.9)	0.43	<.001	0.18	.12
No	87.3	28 (24 to 40)	83.3	28 (25 to 42)	2.9 (1.3 to 9.1)	0.06	.58	0.02	.84
Any drug deductible									
Yes	23.3	48 (36 to 62)	24.5	52 (40 to 70)	17.5 (9.9 to 22.5)	0.25	.03	0.17	.14
No	76.7	26 (23 to 38)	75.5	26 (25 to 40)	1.3 (−1.1 to 9.9)	−0.05	.64	−0.10	.39
Any coinsurance									
Yes	43.2	33 (28 to 52)	44.5	38 (30 to 53)	15.8 (8.6 to 27.5)	0.36	.001	0.00	.99
No	56.8	26 (26 to 38)	55.5	25 (24 to 38)	−0.8 (−3.3 to 1.9)	−0.03	.77	−0.04	.73
Any deductible or coinsurance									
Yes	54.3	34 (28 to 54)	53.7	40 (30 to 54)	15.0 (8.4 to 23.2)	0.38	.001	0.06	.62
No	45.7	26 (26 to 36)	46.3	25 (23 to 35)	−3.3 (−3.3 to 5.0)	−0.09	.45	−0.08	.48

Abbreviations: IQR, interquartile range; OOP, out-of-pocket.

^a^ All prices are in 2017 US dollars.

^b^ In all subgroups, drugs were weighted by use. In the high-deductible plan subgroups, 2 outlier drugs (palbociclib and ponatinib) were excluded from correlation analyses because the change in out-of-pocket spending from 2015 to 2017 exceeded 500%.

**Figure 3.**
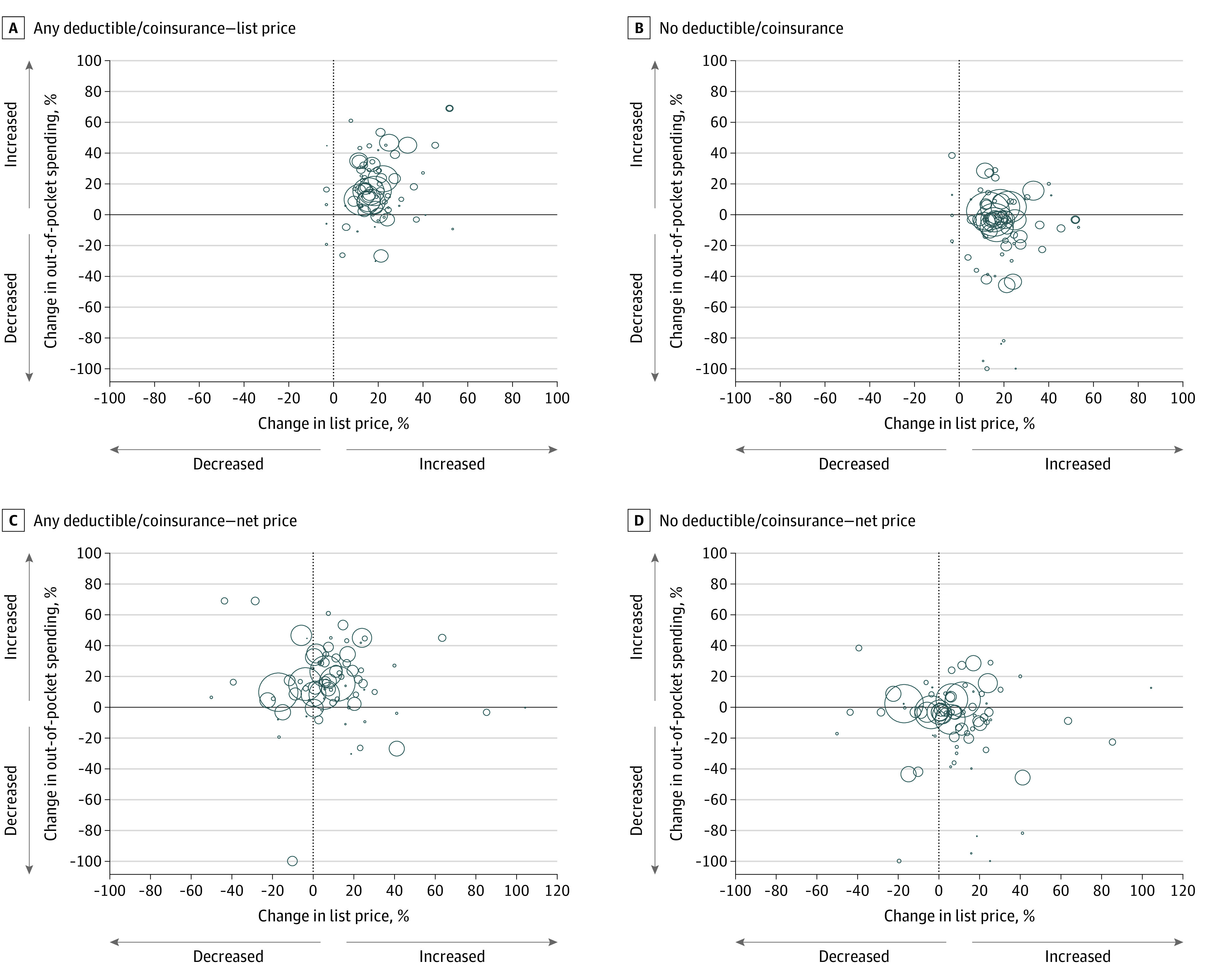
Correlation Between Drug Prices and Out-of-Pocket Spending, by Drug Deductible or Coinsurance Each bubble represents 1 drug, with the area of the bubble proportional to the number of 30-day supplies of the drug dispensed in the US in 2015 and 2017. The x-axis represents the change in list price (panels A and B) or net price (panels C and D), and the y-axis represents the median change in out-of-pocket spending from 2015 to 2017. Panels A and C include patients with any deductible or coinsurance, and panels B and D include patients without any deductible or coinsurance.

Among patients in insurance plans that included deductibles or coinsurance, there was a moderate correlation between changes in out-of-pocket spending and list prices but no correlation with net prices (Figure 3; eFigures 2, 3, and 4 in the Supplement). The strongest correlations were among patients in high-deductible insurance plans (*r* = 0.43; *P* < .001) and those with any deductible or coinsurance (*r* = 0.38; *P* = .001) (Figure 3). Among patients who did not pay any drug deductibles or coinsurance (ie, copayments only), there was no correlation between changes in out-of-pocket spending and list (*r* = 0.09; *P* = .45) or net prices (*r* = 0.08; *P* = .48).

In unweighted analyses, we similarly found a moderate correlation between changes in list prices and out-of-pocket spending among patients with any coinsurance (*r* = 0.30; *P* = .008) and those with any deductible or coinsurance (*r* = 0.24; *P* = .03) (eTable 2 in the Supplement). There was no correlation between changes in net price and out-of-pocket spending. Results were similar when we excluded 21 drugs for which net price estimates exceeded list prices in at least 1 quarter (eTable 3 and eTable 4 in the Supplement).

## Discussion

Among a cohort of commercially insured patients using brand-name prescription drugs, approximately half paid fixed copayments and were insulated from increases in list prices. The other half of patients had prescription drug benefits that included deductibles or coinsurance and, in that cohort, out-of-pocket costs increased when manufacturers increased list prices. Changes in net drug prices accounting for manufacturer rebates were not correlated with changes in patient out-of-pocket spending, suggesting that increasing rebates offered by manufacturers to partially offset list price hikes are not being directly passed on to patients, even if they limit increases to total drug spending.

Earlier studies of the association between changes in drug prices and out-of-pocket costs have yielded mixed results.^10,11^ Our findings suggest that the association is influenced by insurance benefit design, with flat copayments protecting patients from increasing list prices but coinsurance and deductibles exposing patients to these price increases.

In a national survey, 84% of patients with private health insurance had a pharmacy benefit that included 3 or more drug tiers, with more expensive drugs in higher tiers requiring higher out-of-pocket costs.^18^ Traditionally, insurers set flat copayments for each tier, but insurers increasingly rely on coinsurance to limit spending on high-priced drugs.^19^ In 2019, 28% of commercially insured patients faced coinsurance on tier 2 drugs (typically preferred brand-name drugs) and 41% on tier 4 drugs (typically specialty drugs), with coinsurance rates ranging from 24% to 34% of a drug’s list price.^18^ In our study, nearly half of patients paid prescription drug coinsurance.

We found that, for patients whose prescription drug plans included deductibles or coinsurance, increases in a drug’s list price were correlated with higher out-of-pocket costs. This design is problematic because list prices for brand-name drugs, which are unregulated in the US, have long been increasing much faster than inflation.^3^ List prices increased for nearly all drugs in our cohort, with a median inflation-adjusted increase of 16.7% over 2 years. Some of these list price increases have been offset by increasing rebates provided to insurers and pharmacy benefit managers, such that 1 in 4 drugs in our cohort had a decrease in net price from 2015 to 2017. Although increasing rebates may limit the effect of list price hikes on total drug spending, we did not find any correlation between changes in a drug’s net price and out-of-pocket spending, suggesting that rebates are not being reflected in lower point-of-care prices for patients.^4,7^

Tying patient out-of-pocket costs directly to the price of a drug can encourage patients to use less costly medications, and completely detaching patient expenses from drug prices could ultimately lead to higher premiums.^20,21^ However, the parallel unfettered increases in list prices for brand-name drugs and greater reliance by insurers on deductibles and coinsurance have placed important financial burdens on patients using high-cost brand-name medications. Higher out-of-pocket costs have been associated with decreased medication adherence and, in some cases, worse clinical outcomes in a wide range of clinical conditions, including cancer,^22,23^ diabetes,^24,25^ and cardiovascular disease.^26,27,28^ Policy interventions that lower out-of-pocket spending can increase medication adherence, improve clinical outcomes, and reduce health disparities.^14,29,30^

As a result, policy makers should consider how to protect patients from large increases in out-of-pocket spending resulting from year-over-year increases in list prices for prescription drugs. For example, Congress could cap annual increases on list prices or penalize manufacturers for increases above inflation through mandatory rebates in Medicare Part D, similar to the model already used in Medicaid.^31,32^ However, there are concerns that caps or penalties on price increases could encourage manufacturers to set higher prices for drugs at launch,^32^ and if such rules were imposed only on Medicare Part D, there may be no substantial benefit for the half of Americans who are covered by private employer–based health insurance plans.^8^

Policy makers could also address the increasing gap between list and net prices by eliminating confidential manufacturer rebates to insurers and pharmacy benefit managers or mandating that such rebates be passed directly to patients at the point of sale. A rule finalized by the Trump Administration in November 2020 would have eliminated drug rebates in Medicare Part D, although the rule faces substantial legal hurdles and its implementation has been delayed.^33,34^ Lawmakers have proposed more general bans on rebates to public and private payers unless the rebates are directly passed on to patients at the point of sale.^35^ Some private insurers have already begun implementing programs to pass rebates on directly to patients.^36^ However, limiting confidential rebates or requiring increased transparency could undermine the bargaining power of large purchasers, leading to an overall increase in drug prices and spending.^37,38,39^ Thus, a ban on rebates might reduce out-of-pocket spending by patients who use certain high-cost medications but lead to higher premiums among all patients.

### Limitations

This study has limitations. We examined only 79 drugs owing to strict inclusion criteria to ensure accurate measurement of prices, so our findings may not be representative of all brand-name drugs. However, the cohort includes many top-selling drugs, with the entire cohort accounting for $67 billion in spending in 2017, which represents nearly 15% of total US prescription drug spending.^40^ Drugs with multiple formulations or for which monthly price varies by dose are likely to have similar changes over time, although prices and subsequent out-of-pocket spending per patient may be more variable. Because we measured out-of-pocket spending from pharmacy claims, we were unable to include clinician-administered drugs, which account for a growing proportion of prescription drug spending.^41^

Another limitation is that our findings are applicable only to commercially insured patients, which include approximately half of Americans^8^; the association between increasing list prices and increasing out-of-pocket spending may be more pronounced among patients covered by Medicare, as the standard Part D benefit includes coinsurance (25% up until a catastrophic limit, and thereafter 5%).^4,7^ Commercially insured patients may use manufacturer coupons to offset some out-of-pocket spending, and coupon use is not captured in claims data. Coupon use has increased over the past several years,^42,43^ so not being able to account for coupons may lead to an overestimate of out-of-pocket spending for some drugs.

In addition, individual rebates to payers are confidential. The estimates of net prices from SSR Health are based on manufacturer revenue and may underestimate postrebate prescription drug spending by excluding fees and profits among other elements of the pharmaceutical supply chain, including wholesalers, pharmacies, and pharmacy benefit managers.^44,45^ SSR Health may underestimate Medicaid rebates, leading to a corresponding overestimate in estimated non-Medicaid rebates,^46^ although such an overestimate would be unlikely to substantially affect change in net prices over time.

## Conclusions

Although some commercially insured patients who pay only prescription drug copayments are insulated from increases in prescription drug prices, more than half of patients pay deductibles or coinsurance and may experience increases in out-of-pocket spending when manufacturers increase list prices. Among these patients, we found no evidence that increasing manufacturer rebates directly offset increases in out-of-pocket expenses. Policy makers seeking to limit price increases among brand-name drugs should consider how any proposed policies affect patient out-of-pocket spending.

## Notes

Supplement. **eFigure 1.** Drugs in Cohort and Reasons for Exclusion **eTable 1.** Drugs Included in Cohort **eTable 2.** Changes in Out-of-Pocket Spending and Correlation With Changes in Drug Prices, Unweighted **eTable 3.** Drug Prices and Out-of-Pocket Spending, Excluding Drugs With Higher Net Than List Price Estimates in Any Quarter **eTable 4.** Changes in Out-of-Pocket Spending and Correlation With Changes in Drug Prices, Excluding Drugs With Higher Net Than List Price Estimates in Any Quarter **eFigure 2.** Correlation Between Drug Prices and Out-of-Pocket Spending, by High Deductible Insurance Plan **eFigure 3.** Correlation Between Drug Prices and Out-of-Pocket Spending, by Deductible **eFigure 4.** Correlation Between Drug Prices and Out-of-Pocket Spending, by Coinsurance

## References

[1] Schumock GT, Stubbings J, Hoffman JM, . National trends in prescription drug expenditures and projections for 2019. Am J Health Syst Pharm. 2019;76(15):1105-1121. doi:10.1093/ajhp/zxz109 31199861

[2] Hartman M, Martin AB, Benson J, Catlin A; National Health Expenditure Accounts Team. National health care spending in 2018: growth driven by accelerations in Medicare and private insurance spending. Health Aff (Millwood). 2020;39(1):8-17. doi:10.1377/hlthaff.2019.01451 31804875

[3] Hernandez I, San-Juan-Rodriguez A, Good CB, Gellad WF. Changes in list prices, net prices, and discounts for branded drugs in the US, 2007-2018. JAMA. 2020;323(9):854-862. doi:10.1001/jama.2020.1012 32125403 PMC7054846

[4] Dusetzina SB, Bach PB. Prescription drugs—list price, net price, and the rebate caught in the middle. JAMA. 2019;321(16):1563-1564. doi:10.1001/jama.2019.2445 30840047

[5] Roehrig C. Rebates, coupons, PBMs, and the cost of the prescription drug benefit. Health Affairs Blog. April 26, 2018. Accessed March 13, 2021. doi:10.1377/hblog20180424.17957

[6] Dusetzina SB, Keating NL, Huskamp HA. Proposals to redesign Medicare Part D—easing the burden of rising drug prices. N Engl J Med. 2019;381(15):1401-1404. doi:10.1056/NEJMp1908688 31483987

[7] Dusetzina SB, Conti RM, Yu NL, Bach PB. Association of prescription drug price rebates in Medicare part D with patient out-of-pocket and federal spending. JAMA Intern Med. 2017;177(8):1185-1188. doi:10.1001/jamainternmed.2017.1885 28558108 PMC5722464

[8] Kaiser Family Foundation. Health insurance coverage of the total population. 2018. Accessed May 27, 2020. https://www.kff.org/other/state-indicator/total-population/

[9] Kesselheim AS, Avorn J, Sarpatwari A. The high cost of prescription drugs in the United States: origins and prospects for reform. JAMA. 2016;316(8):858-871. doi:10.1001/jama.2016.11237 27552619

[10] Yang EJ, Galan E, Thombley R, . Changes in drug list prices and amounts paid by patients and insurers. JAMA Netw Open. 2020;3(12):e2028510. doi:10.1001/jamanetworkopen.2020.28510 33295971 PMC7726630

[11] Meiri A, Zhang F, Ross-Degnan D, Wharam JF. Trends in insulin out-of-pocket costs and reimbursement price among US patients with private health insurance, 2006-2017. JAMA Intern Med. 2020;180(7):1010-1012. doi:10.1001/jamainternmed.2020.1302 32478823 PMC7265120

[12] SSR Health. US brand Rx net pricing tool. 2019. Accessed March 9, 2020. http://www.ssrhealth.com/us-brand-rx-net-price-tool/

[13] FDB MedKnowledge Drug Database. First Databank. Accessed August 25, 2020. https://www.fdbhealth.com/solutions/medknowledge-drug-database

[14] Pace LE, Dusetzina SB, Keating NL. Early impact of the Affordable Care Act on oral contraceptive cost sharing, discontinuation, and nonadherence. Health Aff (Millwood). 2016;35(9):1616-1624. doi:10.1377/hlthaff.2015.1624 27605641

[15] Centers for Medicare & Medicaid Services. Medicaid drug rebate program. Updated January 22, 2021. Accessed October 26, 2019. https://www.medicaid.gov/medicaid/prescription-drugs/medicaid-drug-rebate-program/index.html

[16] Congressional Budget Office. A comparison of brand-name drug prices among selected federal programs. February 18, 2021. Accessed March 13, 2021. https://www.cbo.gov/publication/56978

[17] Bureau of Labor Statistics. Databases, tables & calculators by subject. 2019. Accessed February 21, 2019. https://www.bls.gov/data/

[18] Kaiser Family Foundation. 2019 Employer health benefits survey, section 9: prescription drug benefits. September 25, 2019. Accessed May 4, 2020. https://www.kff.org/report-section/ehbs-2019-section-9-prescription-drug-benefits/

[19] Lee TH, Emanuel EJ. Tier 4 drugs and the fraying of the social compact. N Engl J Med. 2008;359(4):333-335. doi:10.1056/NEJMp0804261 18650510

[20] Starner CI, Alexander GC, Bowen K, Qiu Y, Wickersham PJ, Gleason PP. Specialty drug coupons lower out-of-pocket costs and may improve adherence at the risk of increasing premiums. Health Aff (Millwood). 2014;33(10):1761-1769. doi:10.1377/hlthaff.2014.0497 25288420

[21] Dafny L, Ody C, Schmitt M. When discounts raise costs: the effect of copay coupons on generic utilization. Am Econ J Econ Policy. 2017;9(2):91-123. doi:10.1257/pol.20150588

[22] Dusetzina SB, Winn AN, Abel GA, Huskamp HA, Keating NL. Cost sharing and adherence to tyrosine kinase inhibitors for patients with chronic myeloid leukemia. J Clin Oncol. 2014;32(4):306-311. doi:10.1200/JCO.2013.52.9123 24366936

[23] Farias AJ, Du XL. Association between out-of-pocket costs, race/ethnicity, and adjuvant endocrine therapy adherence among Medicare patients with breast cancer. J Clin Oncol. 2017;35(1):86-95. doi:10.1200/JCO.2016.68.2807 28034069 PMC5455689

[24] Bibeau WS, Fu H, Taylor AD, Kwan AYM. Impact of out-of-pocket pharmacy costs on branded medication adherence among patients with type 2 diabetes. J Manag Care Spec Pharm. 2016;22(11):1338-1347. doi:10.18553/jmcp.2016.22.11.1338 27783549 PMC10397590

[25] Karter AJ, Parker MM, Solomon MD, . Effect of out-of-pocket cost on medication initiation, adherence, and persistence among patients with type 2 diabetes: the Diabetes Study of Northern California (DISTANCE). Health Serv Res. 2018;53(2):1227-1247. doi:10.1111/1475-6773.12700 28474736 PMC5867086

[26] Choudhry NK, Avorn J, Glynn RJ, ; Post-Myocardial Infarction Free Rx Event and Economic Evaluation (MI FREEE) Trial. Full coverage for preventive medications after myocardial infarction. N Engl J Med. 2011;365(22):2088-2097. doi:10.1056/NEJMsa1107913 22080794

[27] Wang TY, Kaltenbach LA, Cannon CP, . Effect of medication co-payment vouchers on P2Y12 inhibitor use and major adverse cardiovascular events among patients with myocardial infarction: the ARTEMIS randomized clinical trial. JAMA. 2019;321(1):44-55. doi:10.1001/jama.2018.19791 30620370 PMC6583585

[28] Rome BN, Gagne JJ, Avorn J, Kesselheim AS. Non-warfarin oral anticoagulant copayments and adherence in atrial fibrillation: a population-based cohort study. Am Heart J. 2021;233:109-121. doi:10.1016/j.ahj.2020.12.010 33358690

[29] Lewey J, Shrank WH, Avorn J, Liu J, Choudhry NK. Medication adherence and healthcare disparities: impact of statin co-payment reduction. Am J Manag Care. 2015;21(10):696-704. https://www.ncbi.nlm.nih.gov/pubmed/26633094. 26633094

[30] Chernew ME, Shah MR, Wegh A, . Impact of decreasing copayments on medication adherence within a disease management environment. Health Aff (Millwood). 2008;27(1):103-112. doi:10.1377/hlthaff.27.1.103 18180484

[31] Dusetzina SB, Oberlander J. Advancing legislation on drug pricing—is there a path forward? N Engl J Med. 2019;381(22):2081-2084. doi:10.1056/NEJMp1914044 31774951

[32] Anderson-Cook A, Love K, Noda A, Miller ME. How a Medicare Part D inflation penalty would lower drug spending for patients, taxpayers, and employers. Health Affairs Blog. February 5, 2020. Accessed March 13, 2021. doi:10.1377/hblog20200204.864372

[33] Sachs R. Trump Administration releases long-awaited drug rebate proposal. Health Affairs Blog. February 1, 2019. Accessed May 13, 2020. https://www.healthaffairs.org/do/10.1377/hblog20190201.545950/full/

[34] Sachs R. Administration finalizes drug pricing rebate rule at the last minute. Health Affairs Blog. November 23, 2020. Accessed March 2, 2021. https://www.healthaffairs.org/do/10.1377/hblog20201122.985836/full/

[35] Congess.gov. S.657—a bill to amend Title XXVII of the Public Health Service Act to establish requirements with respect to prescription drug benefits, 116th Congress (2019-2020). Accessed March 13, 2021. https://www.congress.gov/bill/116th-congress/senate-bill/657

[36] Johnson CY. UnitedHealthcare will provide drug rebates directly to members in some plans. *The Washington Post*. March 6, 2018. Accessed May 13, 2020. https://www.washingtonpost.com/news/wonk/wp/2018/03/06/unitedhealthcare-will-provide-drug-rebates-directly-to-members-in-some-plans/

[37] Gellad WF, Ennis M, Kuza CC. A new safe harbor—turning drug rebates into discounts. N Engl J Med. 2019;380(18):1688-1690. doi:10.1056/NEJMp1902692 30946552

[38] Congressional Budget Office. incorporating the effects of the proposed rule on safe harbors for pharmaceutical rebates in CBO’s budget projections—supplemental material for updated budget projections: 2019 to 2029. May 2019. Accessed March 13, 2021. https://www.cbo.gov/system/files/2019-05/55151-SupplementalMaterial.pdf

[39] Seeley E, Kesselheim AS. Pharmacy benefit managers: practices, controversies, and what lies ahead. The Commonwealth Fund. March 26, 2019. Accessed July 1, 2020. https://www.commonwealthfund.org/publications/issue-briefs/2019/mar/pharmacy-benefit-managers-practices-controversies-what-lies-ahead 30990594

[40] Aitken M, Keinrock M, Campanelli G, Tawil C, Vokey M. Medicine spending and affordability in the US: understanding patients’ costs for medicines. August 4, 2020. Accessed March 31, 2021. https://www.iqvia.com/insights/the-iqvia-institute/reports/medicine-spending-and-affordability-in-the-us

[41] Hwang TJ, Jain N, Lauffenburger JC, Vokinger KN, Kesselheim AS. Analysis of proposed Medicare Part B to Part D shift with associated changes in total spending and patient cost-sharing for prescription drugs. JAMA Intern Med. 2019;179(3):374-380. doi:10.1001/jamainternmed.2018.6417 30640379 PMC6439690

[42] Massachusetts Health Policy Commission. Prescription drug coupon study: report to the Massachusetts Legislature. Updated August 14, 2020. Accessed March 13, 2021. https://www.mass.gov/doc/prescription-drug-coupon-study

[43] IQVIA Institute for Human Data Science. Medicines use and spending in the US: a review of 2016 and outlook to 2021. May 4, 2017. Accessed March 31, 2021. https://www.iqvia.com/institute/reports/medicines-use-and-spending-in-the-us-a-review-of-2016

[44] Yu NL, Atteberry P, Bach PB. Spending on prescription drugs in the US: where does all the money go? Health Affairs Blog. July 31, 2018. Accessed May 27, 2020. https://www.healthaffairs.org/do/10.1377/hblog20180726.670593/full/

[45] Rome BN, Feldman WB, Kesselheim AS. Drug prices, rebates, and discounts. JAMA. 2020;324(4):399. doi:10.1001/jama.2020.7983 32720997

[46] Anderson-Cook A, Noda A. Drug prices, rebates, and discounts. JAMA. 2020;324(4):398-399. doi:10.1001/jama.2020.7989 32720999

