# Supplementary material for: Correlation Between Changes in Brand-Name Drug Prices and Patient Out-of-Pocket Costs
Source: JAMA Netw Open. 2021 May 4;4(5):e218816. doi: 10.1001/jamanetworkopen.2021.8816 (PMC8097497; doi:10.1001/jamanetworkopen.2021.8816)
Supplement: Supplement. — eFigure 1. Drugs in Cohort and Reasons for Exclusion eTable 1. Drugs Included in Cohort eTable 2. Changes in Out-of-Pocket Spending and Correlation With Changes in Drug Prices, Unweighted eTable 3. Drug Prices and Out-of-Pocket Spending, Excluding Drugs With Higher Net Than List Price Estimates in Any Quarter eTable 4. Changes in Out-of-Pocket Spending and Correlation With Changes in Drug Prices, Excluding Drugs With Higher Net Than List Price Estimates in Any Quarter eFigure 2. Correlation Between Drug Prices and Out-of-Pocket Spending, by High Deductible Insurance Plan eFigure 3. Correlation Between Drug Prices and Out-of-Pocket Spending, by Deductible eFigure 4. Correlation Between Drug Prices and Out-of-Pocket Spending, by Coinsurance [file jamanetwopen-e218816-s001.pdf]

Supplementary Online Content

Rome BN, Feldman WB, Desai RJ, Kesselheim AS. Correlation between changes in brand-name drug prices and patient out-of-pocket costs. *JAMA Netw Open*. 2021;4(5):e218816. doi:10.1001/jamanetworkopen.2021.8816

eFigure 1. Drugs in Cohort and Reasons for Exclusion

eTable 1. Drugs Included in Cohort

eTable 2. Changes in Out-of-Pocket Spending and Correlation With Changes in Drug Prices, Unweighted

eTable 3. Drug Prices and Out-of-Pocket Spending, Excluding Drugs With Higher Net Than List Price Estimates in Any Quarter

eTable 4. Changes in Out-of-Pocket Spending and Correlation With Changes in Drug Prices, Excluding Drugs With Higher Net Than List Price Estimates in Any Quarter

eFigure 2. Correlation Between Drug Prices and Out-of-Pocket Spending, by High Deductible Insurance Plan

eFigure 3. Correlation Between Drug Prices and Out-of-Pocket Spending, by Deductible

eFigure 4. Correlation Between Drug Prices and Out-of-Pocket Spending, by Coinsurance

This supplementary material has been provided by the authors to give readers additional information about their work.

© 2021 Rome BN et al. *JAMA Network Open.*

eFigure 1: Drugs in Cohort and Reasons for Exclusion

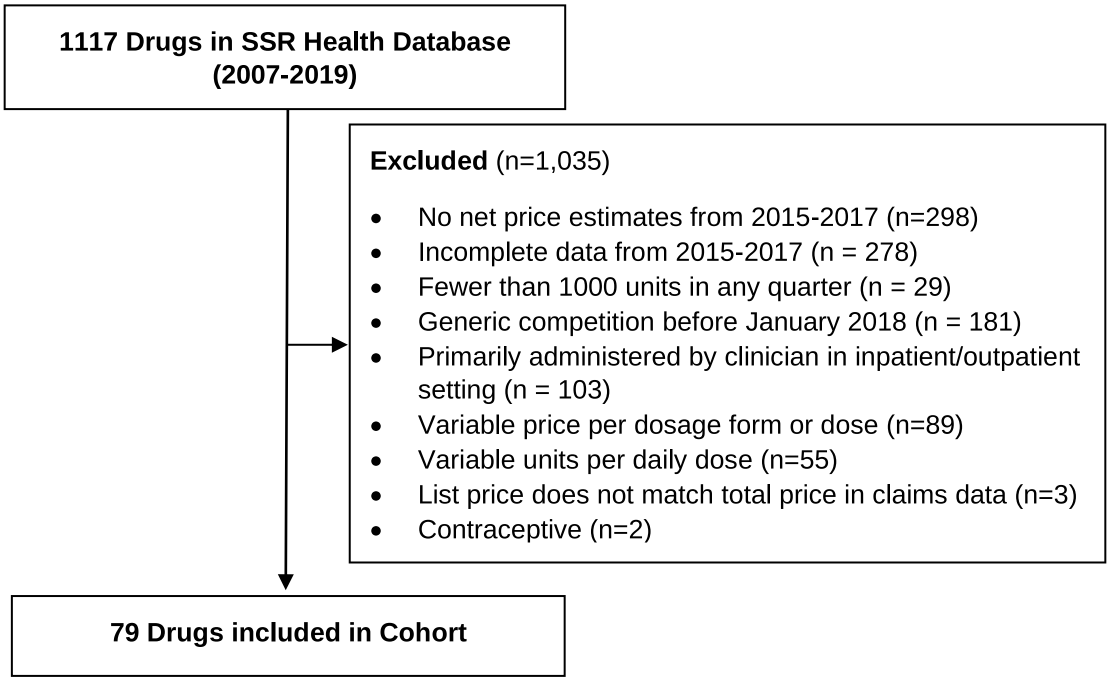

© 2021 Rome BN et al. *JAMA Network Open.*

eTable 1: Drugs included in Cohort

Generic Name	Brand Name	Route of Administration	Therapeutic Category	**US 30-Day Supplies, 2015 and 2017** (Thousands)
Sitagliptin	Januvia	Oral	Endocrine / Diabetes	28,446
Lisdexamfetamine	Vyvanse	Oral	Psychiatric	24,002
Rivaroxaban	Xarelto	Oral	Cardiovascular	20,906
Nebivolol	Bystolic	Oral	Cardiovascular	19,192
Apixaban	Eliquis	Oral	Cardiovascular	17,151
Dexlansoprazole	Dexilant	Oral	Gastrointestinal	11,400
Solifenacin	Vesicare	Oral	Genitourinary	7,910
Adalimumab	Humira	Injection	Other	7,036
Linagliptin	Tradjenta	Oral	Endocrine / Diabetes	6,999
Linaclotide	Linzess	Oral	Gastrointestinal	6,037
Mirabegron	Myrbetriq	Oral	Genitourinary	5,582
Emtricitabine, Tenofovir	Truvada	Oral	Antimicrobial	4,540
Dulaglutide	Trulicity	Injection	Endocrine / Diabetes	4,512
Memantine	Namenda XR	Oral	Neurologic	4,466
Empagliflozin	Jardiance	Oral	Endocrine / Diabetes	4,310
Febuxostat	Uloric	Oral	Musculoskeletal	3,852
Ticagrelor	Brilinta	Oral	Cardiovascular	3,572
Vilazodone	Viibryd	Oral	Psychiatric	3,367
Saxagliptin	Onglyza	Oral	Endocrine / Diabetes	3,355
Lubiprostone	Amitiza	Oral	Gastrointestinal	2,886
Vortioxetine	Trintellix	Oral	Psychiatric	2,855
Silodosin	Rapaflo	Oral	Genitourinary	2,298
Efavirenz, Emtricitabine, Tenofovir	Atripla	Oral	Antimicrobial	2,263
Umeclidinium, Vilanterol	Anoro Ellipta	Inhalation	Respiratory	2,244
Fesoterodine	Toviaz	Oral	Genitourinary	1,820
Dronedarone	Multaq	Oral	Cardiovascular	1,793
Polyethylene glycol 3350, Electrolytes	Moviprep	Oral	Gastrointestinal	1,739
Abacavir, Dolutegravir. Lamivudine	Triumeq	Oral	Antimicrobial	1,547
Roflumilast	Daliresp	Oral	Respiratory	1,360
Elvitegravir, Cobicistat, Emtricitabine, Tenofovir	Stribild	Oral	Antimicrobial	1,265
Mesalamine	Apriso	Oral	Gastrointestinal	1,259
Milnacipran	Savella	Oral	Musculoskeletal	1,148
Ibuprofen, Famotidine	Duexis	Oral	Musculoskeletal	1,140
Dimethyl Fumarate	Tecfidera	Oral	Neurologic	1,100
Teriparatide	Forteo	Injection	Endocrine / Diabetes	998
Emtricita, Rilpivirine, Tenofovir	Complera	Oral	Antimicrobial	870
Levomilnacipran	Fetzima	Oral	Psychiatric	808
Albiglutide	Tanzeum	Injection	Endocrine / Diabetes	786
Naproxen, Esomeprazole	Vimovo	Oral	Musculoskeletal	781
Ledipasvir, Sofosbuvir	Harvoni	Oral	Antimicrobial	682
Empagliflozin, Linagliptin	Glyxambi	Oral	Endocrine / Diabetes	595
Fingolimod	Gilenya	Oral	Neurologic	564
Mesalamine	Canasa	Rectal	Gastrointestinal	564
Pegfilgrastim	Neulasta	Injection	Oncology	552

© 2021 Rome BN et al. *JAMA Network Open.*

Generic Name	Brand Name	Route of Administration	Therapeutic Category	US 30-Day Supplies, 2015 and 2017 (Thousands)
Asenapine	Saphris	Oral	Psychiatric	524
Dalfampridine	Ampyra	Oral	Neurologic	523
Arformoterol	Brovana	Inhalation	Respiratory	513
Fosfomycin Tromethamine	Monurol	Oral	Antimicrobial	475
Rotigotine	Neupro	Topical	Neurologic	463
Dornase Alfa	Pulmozyme	Inhaled	Respiratory	387
Choriogonadotropin Alfa	Ovidrel	Injection	Endocrine / Diabetes	361
Dextromethorphan, Quinidine	Nuedexta	Oral	Neurologic	355
Teriflunomide	Aubagio	Oral	Neurologic	342
Palbociclib	Ibrance	Oral	Oncology	311
Enzalutamide	Xtandi	Oral	Oncology	290
Nilotinib	Tasigna	Oral	Oncology	259
Interferon Beta-1B	Betaseron	Injection	Neurologic	190
Tadalafil	Adcirca	Oral	Genitourinary	167
Ambrisentan	Letairis	Oral	Cardiovascular	162
Rilpivirin	Edurant	Oral	Antimicrobial	142
Pazopanib	Votrient	Oral	Oncology	140
Sofosbuvir	Sovaldi	Oral	Antimicrobial	129
Vitamin B Complex, Folic Acid, Vitamin C, Biotin	Nephro-Vite Rx	Oral	Other	122
Sodium Phosphate	Osmoprep	Oral	Gastrointestinal	116
Pomalidomide	Pomalyst	Oral	Oncology	110
Bismuth, Metronidazole, Tetracycline	Pylera	Oral	Gastrointestinal	91
Macitentan	Opsumit	Oral	Cardiovascular	84
Diclofenac	Zipsor	Oral	Musculoskeletal	80
Ruxolitinib	Jakafi	Oral	Oncology	49
Oxybutynin	Oxytrol	Topical	Genitourinary	47
Peginterferon Beta-1A	Plegridy	Injection	Neurologic	45
Crizotinib	Xalkori	Oral	Oncology	45
Hydroxypropyl Cellulose	Lacrisert	Ophthalmic	Other	44
Ivacaftor	Kalydeco	Oral	Respiratory	31
Ponatini	Iclusig	Oral	Oncology	29
Sorafenib	Nexavar	Oral	Oncology	27
Bosentan	Tracleer	Oral	Cardiovascular	23
Regorafenib	Stivarga	Oral	Oncology	18
Eliglustat Tartrate	Cerdelga	Oral	Other	8

Legend: The table lists all 79 drugs included in the analysis. The route of administration and therapeutic category, based on the drug's label, are shown for each drug. The drugs are sorted in decreasing order of use (final column), defined as the number of 30-day supplies dispensed in the US in 2015 and 2017. This utilization figure was used to weight drugs in the main analysis.

© 2021 Rome BN et al. *JAMA Network Open.*

eTable 2: Changes in Out-of-Pocket Spending and Correlation with changes in Drug Prices, Unweighted

**Subgroup**	**2015**			**2017**		**Percent Change in OOP Spending, 2015 to 2017,** Median (IQR)	**Correlation with Change in List Price**			**Correlation with Change in Net Price**	
	**Patients (%)**	**OOP Spending;** Median (IQR)		**Patients (%)**	**OOP Spending;** Median (IQR)		**r**	**P-value**		**r**	**P-Value**
**All Patients**	100	36 (31, 47)		100	40 (30, 50)	4.9 (-3.3, 14.7)	0.07	0.56		-0.08	0.47
**High Deductible Plan***											
Yes	12.7	62 (49, 83)		16.7	65 (51, 100)	7.8 (-5.6, 26.6)	0.17	0.13		-0.10	0.41
No	87.3	36 (28, 45)		83.3	35 (29, 47)	2.9 (-3.3, 10.5)	0.02	0.89		-0.16	0.15
**Any Drug Deductible**											
Yes	23.3	59 (48, 92)		24.5	75 (47, 125)	18.3 (3.3, 5.2)	0.05	0.69		-0.01	0.93
No	76.7	31 (26, 41)		75.5	33 (27, 45)	**2.9 (-3.3, 14.3)**	-0.05	0.69		-0.08	0.47
**Any Coinsurance**											
Yes	43.2	41 (30, 59)		44.5	46 (37, 65)	**15.8 (2.9, 28.9)**	**0.30**	**0.008**		-0.06	0.61
No	56.8	36 (26, 43)		55.5	34 (25, 46)	-3.3 (-14.3, 7.5)	-0.11	0.34		-0.12	0.29
**Any Deductible or Coinsurance**											
Yes	54.3	42 (32 (620		53.7	50 (39, 69)	15.0 (4.4, 28.9)	**0.24**	**0.03**		-0.07	0.57
No	45.7	34 (26, 41)		46.3	30 (25, 40)	-3.3 (-17.2, 6.6)	0.01	0.95		-0.07	0.52

Abbreviations: IQR = Interquartile range; OOP = Out-of-Pocket

* In the high-deductible plan subgroups, 2 outlier drugs (Palbociclib and Ponatinib) were excluded from correlation analyses because the change in out-of-pocket spending from 2015 to 2017 exceeded 500%.

Legend: The first 4 columns show the percent of patients in each subgroup and the median out-of-pocket spending for each drug among patients in that subgroup, weighted by drug utilization. All spending figures are in 2017 US dollars and represent the median spending for a 30-day drug supply among the given cohort. The next column shows the relative change in out-of-pocket spending from 2015 to 2017 among each subgroup. The final 4 columns show the correlation between change in drug list/net prices and out-of-pocket spending. Significant correlations ($p < 0.05$) are bolded.

© 2021 Rome BN et al. *JAMA Network Open.*

eTable 3: Drug Prices and Out-of-Pocket Spending, Excluding Drugs with Higher Net than List Price Estimates in Any Quarter

	**Unweighted**		
	**2015 Price per 30-day Supply;** median (IQR)	**2017 Price per 30-day Supply (USD);** median (IQR)	**Percent Change, 2015 to 2017;** median (IQR)
**List Price**	453 (273, 2236)	537 (328, 2589)	17.7 (12.8, 24.1)
**Net Price**	268 (166, 1397)	236 (169, 1643)	6.3 (-3.7, 16.9)
**Out-of-Pocket Spending**	38 (29, 49)	40 (30, 54)	5.3 (-1.3, 14.3)
	**Weighted by Drug Use**		
**List Price**	333 (229, 346)	385 (271, 390)	16.7 (13.6, 21.1)
**Net Price**	173 (128, 223)	166 (144, 219)	2.7 (-3.9, 11.2)
**Out-of-Pocket Spending**	29 (26, 43)	30 (26, 45)	3.5 (1.4, 9.1)

Abbreviations: IQR = Interquartile Range

Legend: All prices represent the cost of a 30-day drug supply and are in 2017 US dollars. Results are weighted by drug use, defined as the number of 30-day supplies dispensed in the US in 2015 and 2017.

© 2021 Rome BN et al. *JAMA Network Open.*

eTable 4: Changes in Out-of-Pocket Spending and Correlation with Changes in Drug Prices, Excluding Drugs with Higher Net than List Price Estimates in Any Quarter

Subgroup	2015		2017		Percent Change in OOP Spending, 2015 to 2017, weighted median (IQR)	Weighted Correlation with Change in List Price		Weighted Correlation with Change in Net Price	
	Patients (%)	OOP Spending; Weighted Median (IQR)	Patients (%)	OOP Spending; Weighted Median (IQR)		r	P-value	r	P-Value
**All Patients**	100	29 (26, 43)	100	30 (26, 45)	3.5 (1.4, 9.1)	0.14	0.29	0.03	0.82
**High Deductible Plan***									
Yes	12.7	55 (51, 66)	16.7	56 943, 65)	-3.3 (-6.2, 8.9)	**0.47**	**<0.001**	0.20	0.13
No	87.3	28 (24, 40)	83.3	28 (25, 42)	2.9 (1.3, 9.1)	0.06	0.67	0.00	0.98
**Any Drug Deductible**									
Yes	23.3	48 (36, 62)	24.5	52 (40, 69)	17.5 (9.9, 22.5)	**0.27**	**0.04**	0.17	0.19
No	76.7	26 (23, 38)	75.5	26 (25, 40)	1.3 (-1.1, 9.9)	-0.07	0.63	-0.12	0.35
**Any Coinsurance**									
Yes	43.2	33 (28, 52)	44.4	38 (30, 53)	15.8 (8.6, 27.5)	**0.38**	**0.003**	-0.00	0.97
No	56.8	26 (26, 38)	55.6	25 (24, 38)	-0.8 (-3.3, 1.9)	-0.05	0.72	-0.06	0.65
**Any Deductible or Coinsurance**									
Yes	54.3	34 (28, 54)	53.6	40 (30, 55)	15.0 (8.4, 23.2)	**0.40**	**0.002**	0.05	0.71
No	45.7	26 (24, 36)	46.4	25 (23, 35)	-3.3 (-3.3, 5.0)	-0.11	0.40	-0.10	0.43

Abbreviations: IQR = Interquartile range; OOP = Out-of-Pocket

* In the high-deductible plan subgroups, 2 outlier drugs (palbociclib and ponatinib) were excluded from correlation analyses because the change in out-of-pocket spending from 2015 to 2017 exceeded 500%.

Legend: The first 4 columns show the percent of patients in each subgroup and the median out-of-pocket spending for each drug among patients in that subgroup, weighted by drug utilization. All spending figures are in 2017 US dollars and represent the median spending for a 30-day drug supply among the given cohort. The next column shows the relative change in out-of-pocket spending from 2015 to 2017 among each subgroup. The final 4 columns show the correlation between change in drug list/net prices and out-of-pocket spending. Significant correlations ($p < 0.05$) are bolded. In all subgroups, drugs are weighted by use.

© 2021 Rome BN et al. *JAMA Network Open.*

eFigure 2: Correlation between Drug Prices and Out-of-Pocket Spending, by High Deductible Insurance Plan

A. High Deductible Insurance Plan **B. Not High Deductible Insurance Plan**

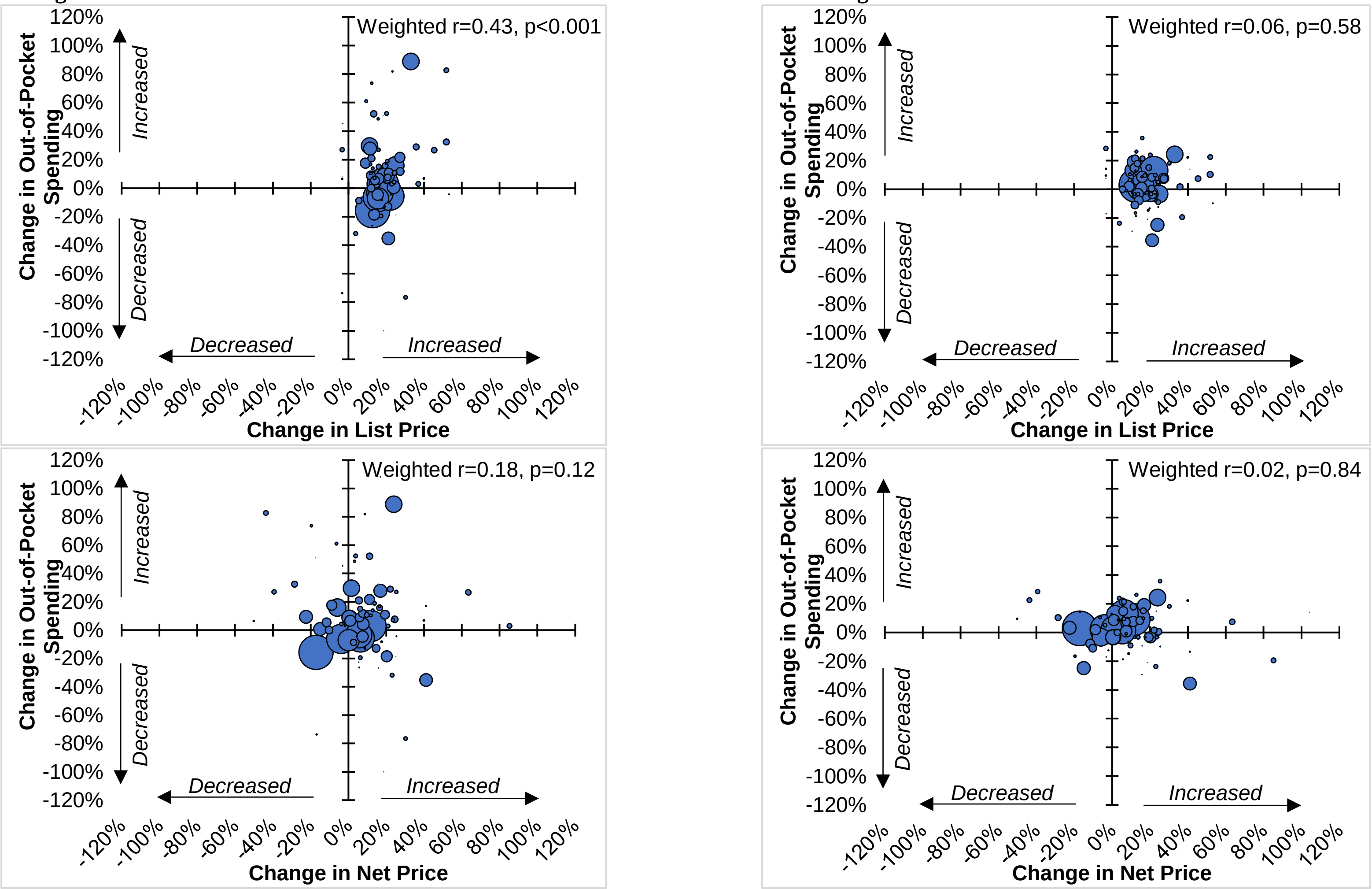

Legend: Each bubble represents one drug, with the area of the bubble proportional to the drug's use (number of 30-day supplies of the drug dispensed in the US in 2015 and 2017). The x-axis represents the change in list or net price, and the y-axis represents the median change in out-of-pocket spending from 2015 to 2017. Panel A includes patients with a high-deductible health plan, and Panel B includes patients without a high-deductible plan (B).

© 2021 Rome BN et al. *JAMA Network Open.*

eFigure 3: Correlation between Drug Prices and Out-of-Pocket Spending, by Deductible

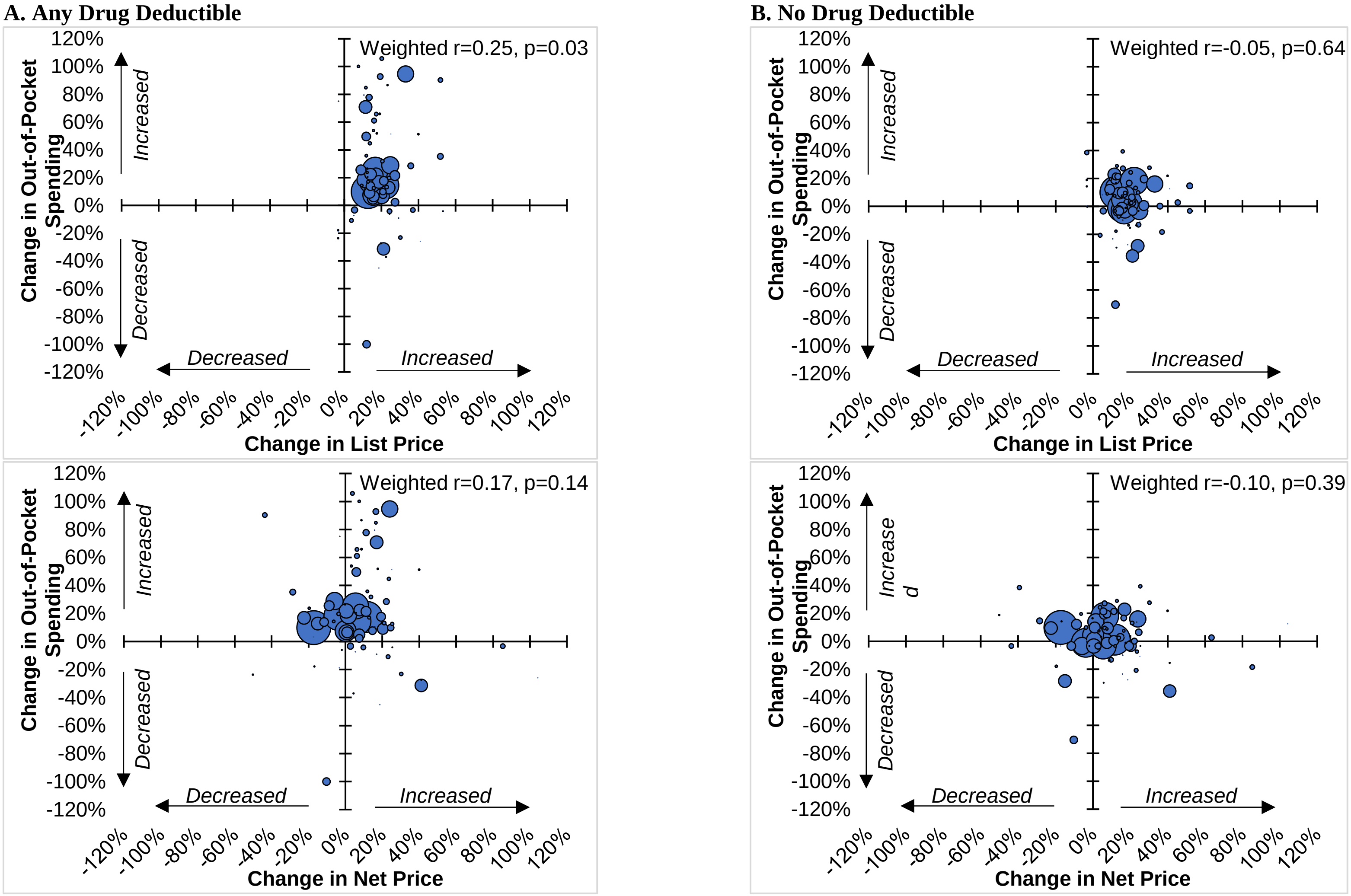

Legend: Each bubble represents one drug, with the area of the bubble proportional to the drug's use (number of 30-day supplies of the drug dispensed in the US in 2015 and 2017). The x-axis represents the change in list or net price, and the y-axis represents the median change in out-of-pocket spending from 2015 to 2017. Panel A includes patients with a drug deductible, and Panel B includes patients without any drug deductible.

© 2021 Rome BN et al. *JAMA Network Open.*

eFigure 4: Correlation between Drug Prices and Out-of-Pocket Spending, by Coinsurance

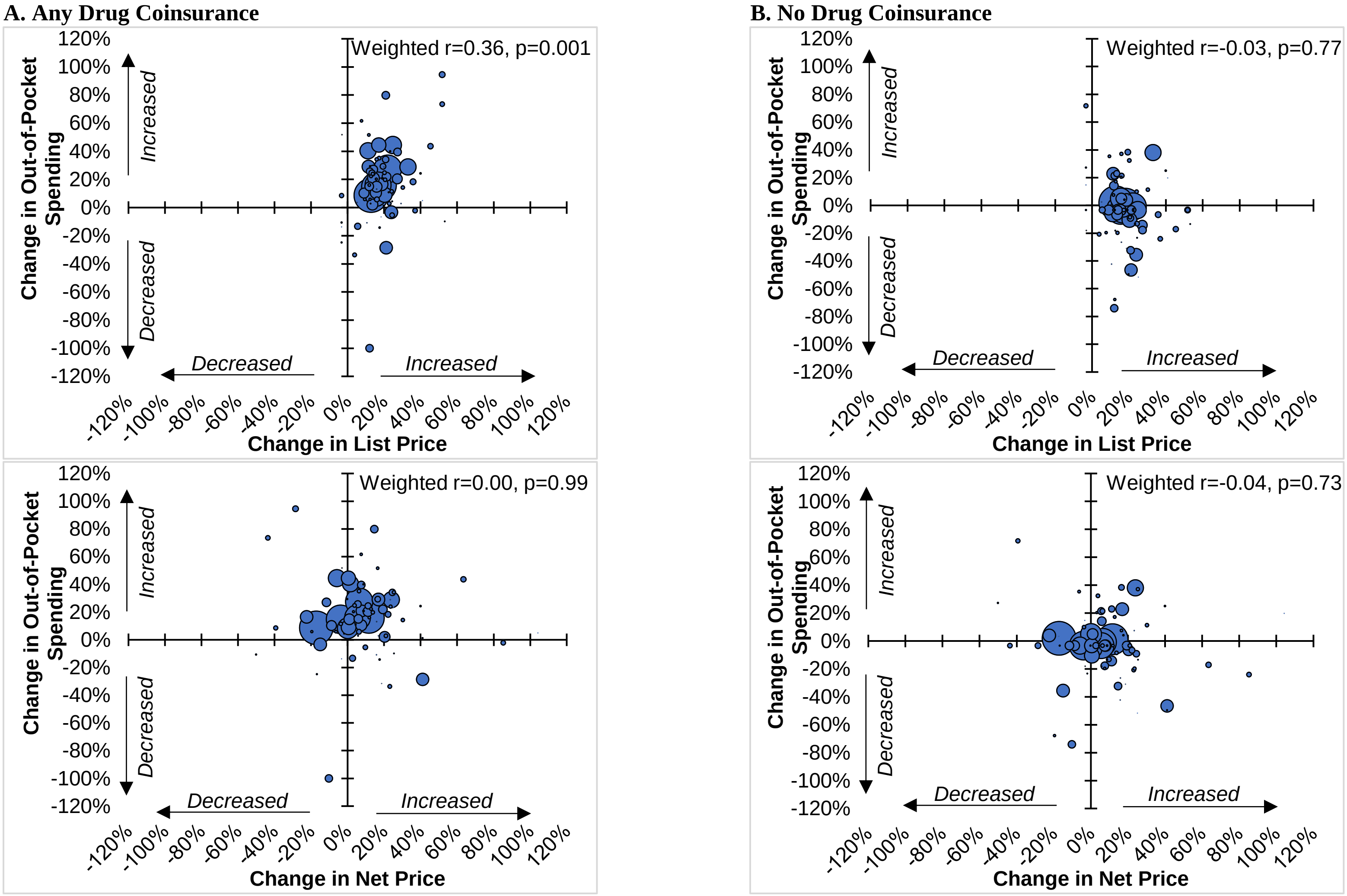

Legend: Each bubble represents one drug, with the area of the bubble proportional to the drug's use (number of 30-day supplies of the drug dispensed in the US in 2015 and 2017). The x-axis represents the change in list or net price, and the y-axis represents the median change in out-of-pocket spending from 2015 to 2017. Panel A includes patients with coinsurance, and Panel B includes patients without any coinsurance.

© 2021 Rome BN et al. *JAMA Network Open.*
